# A commentary on the medicinal use of marijuana

**DOI:** 10.1186/s41182-019-0161-x

**Published:** 2019-05-24

**Authors:** Gehad Mohamed Tawfik, Mohammad Rashidul Hashan, Abdelaziz Abdelaal, Thuan Minh Tieu, Nguyen Tien Huy

**Affiliations:** 1 Faculty of Medicine, Ain Shams University, Cairo, Egypt; 2 http://www.onlineresearchclub.org; 3 Respiratory and Enteric Infections Department, Infectious Disease Division, International Centre for Diarrheal Disease Research, Dhaka, Bangladesh; 4 Faculty of Medicine, Tanta University, Gharbia, Egypt; 5 Faculty of Health Sciences, McMaster University, Hamilton, ON Canada; 6 Department of Clinical Product Development, Institute of Tropical Medicine (NEKKEN), Graduate School of Biomedical Sciences, Nagasaki University, 1-12-4 Sakamoto, Nagasaki, 852-8523 Japan

**Keywords:** Marijuana, Medical, Pain, Treatment, Patients, PTSD

## Abstract

**Background:**

Lately, the number of people using marijuana in the USA has dramatically increased. In 2018, many states have legalized marijuana use for both medical and recreational purposes, thus exploring the evidence behind medical marijuana use became essential. Muslim majority countries enforce rigorous rules as marijuana has been a long-debated issue due to the stigma associated with its use as a treatment. Marijuana has a high beneficial effect in managing chronic pain in adults and relieving spasticity symptoms in multiple sclerosis, obstructive sleep apnea syndrome, and fibromyalgia. As well as, used as pain management, and as anti-emetic in treatment of chemotherapy-induced vomiting and nausea. Marijuana is requested from more than one-third of posttraumatic stress disorder patients due to its significant clinical improvement in nightmares and subsidence disorder symptoms.

Marijuana adversely affects the body’s resistance to many infections, compromising their immune response. Its recreational use has led to an increasing trend in the occurrence of major acute cardiovascular events as stroke, epilepsy, acute myocardial infarction, congestive heart failure, and arrhythmia.

**Conclusion:**

Many countries started to allow medicinal use of marijuana due to its beneficial effect in managing chronic pain, spasticity symptoms in multiple sclerosis, obstructive sleep apnea syndrome, fibromyalgia, and posttraumatic stress disorder. But literature lacks benefit-harm analysis for marijuana usage in medicine. Therefore, evidence-based report of short- and long-term health effects of marijuana use—both harmful and beneficial effects—is crucial for further marijuana prescription in healthcare settings.

Medicinal marijuana is legally used in the USA in 33 states and the District of Columbia, while other 10 states allow consuming it for both recreational and medical proposes [1]. In 2018, marijuana legislation is being considered in extra 12 states. Other than the USA, Uruguay is the first country to fully legalize marijuana in 2013, followed by Canada in 2018. World Health Organization reported that about 2.5% of the world’s population—147 million people—are using marijuana, 10 times more than cocaine or opiates [2]. About 45–80% of patients have prescribed marijuana for managing their chronic pain conditions. It is even used in conjunction with a long-term opioid therapy in 39% patients [3].

Currently, Muslim majority countries enforce rigorous rules as marijuana has been a long debated issue, but there are countries such as the Islamic Republic of Iran where reformation is under consideration. Experience of medical marijuana with counterintuitive religious interpretations has led policymakers to review legal decisions in countries as Iran, yet to implement on national perspective [4]. Noteworthy, a pattern has been noted in the use of marijuana of its various forms (smoke, vapor, edible, concentrates, and topical) in different parts of the world where marijuana has been legalized either for medical or recreational purposes. A recently published national survey in the USA reported that 14.6% of the total population has used marijuana in the past years, while 8.7% of them used it in the past month. Moreover, states where recreational marijuana use has been legalized, have shown the highest incidence of marijuana smoke of 16%. On the other hand, only 11.4% has smoked marijuana in states where it was not legalized [5].

Medicinal marijuana has been approved for the treatment of diseases like HIV/AIDS, cancer, glaucoma, and posttraumatic stress disorder. In 2016, it was reported that the usage of medicinal marijuana helped head and neck cancer patients with long-term side effects of radiotherapy [6].

However, there is an alarming rise in cardiovascular and respiratory problems after its consumption. A CARDIA meta-analysis study reported that 38% of its 3500 participants used marijuana with a positive correlation between its usage, hypertension, and dyslipidemia, which may lead to coronary artery disease [7]. Impact of marijuana usage on the cardiovascular system (CVS) is still unclear, due to the small sample size of patients with acute myocardial infarction (AMI) used in those studies. However, Frost et al. in their study aimed to study long-term outcomes in marijuana users post-AMI and found no association between its consumption and long-term mortality in 2097 post-AMI patients with 109 marijuana users followed up to 18 years [8]. Recently in 2017, Desai et al. analyzed big data of patients (500,000), which reported 3 ± 8% increase in lifetime odds of AMI in marijuana users, but with no significant raise in-hospital mortality, compared to AMI in non-marijuana users [9]. A critical remark, that 1.2 million regular marijuana users for recreational in France reported approximately 2000 adverse events which considered a remarkably low complication rate, maybe due to under-reporting of marijuana use [10]. To further elucidate on this perspective, analyzed data from National Inpatient Sample (NIS) database showed males had a higher prevalence of arrhythmia compared to female counterpart due to usage of marijuana and all-cause inpatient mortality rate among hospitalized marijuana users with arrhythmia too had 15.9% of relative increased risk [11]. It has also been reported that such recreational use of marijuana has led to the increasing trend in the occurrence of major acute cerebrovascular and cardiovascular events especially stroke, epilepsy, acute myocardial infarction, congestive heart failure, and almost all subtypes of arrhythmia [12]. As most often illicit substance users are exposed to a variety of harmful agents like smoking, alcohol abuse, amphetamine or cocaine, consequently large Swedish cohort study did not find any significant association of incidence of early stroke among marijuana users when these potential confounders were adjusted [13]. This recent evidence conflicts with Westover et al. where researchers found ischemic stroke was associated with marijuana abuse and dependence [14]. These findings emphasize the necessity for further evaluation of the effect of marijuana usage on the CVS putting into consideration, its medical usage and doses, not a recreational point of view.

Contradictory findings are reported in literature regarding recommending or prohibiting the use of marijuana for various medical conditions.

In addition to Marijuana’s mentioned medical benefits, it is linked to various adverse effects regarding short-term use and long-term use. For short-term use, it may lead to disturbance in the short-term memory and cognitive functions; not only that but also psychosis in high doses [15]. Addiction to marijuana is connected to long-term use [16]. Furthermore; Marijuana’s heavy users showed slow brain development, lower IQ, symptoms of chronic bronchitis and chronic psychosis [17]. The association between cancer and marijuana use is under investigation; as studies on head and neck cancers reported contradicting results, another lung cancer studies supported no association, but some studies succeeded in linking between testicular cancer and marijuana usage [18].

Despite the lack of a thorough benefit-harm analysis, marijuana has proven effective for pain management, and as anti-emetic in the treatment of chemotherapy-induced vomiting and nausea. Synthetic marijuana analog such as nabilole, levonantradol, and tetrahydrocannabinol reported mildly effective in cancer chemotherapy emesis palliation [19]. Ironically, these agents were not compared to the newly available anti-emetic agents and also narrow therapeutic window demands careful titration of dosage which further limits its usage [20]. It also reduces patient-reported spasticity symptoms in patients with multiple sclerosis [21]. Surprisingly, a recent meta-analysis of 19 randomized clinical trials of moderate quality (11 trials of high risk of bias; 6 trials of unknown risk of bias; 2 trials of low risk of bias) concluded that cannabinoids are beneficial in the treatment of obstructive sleep apnea syndrome (two trials), fibromyalgia (two trials), chronic pain (eight trials), and multiple sclerosis (seven trials) [22]. On the other hand, there is insufficient evidence to be able to draw conclusions regarding therapeutic effects of marijuana use in various medical conditions, some of which are anxiety symptoms in individuals with social anxiety disorders, mortality, or disability in patients after traumatic brain injury or intracranial hemorrhage, glaucoma, cancers, motor symptoms associated with Parkinson’s disease, epilepsy, and mental health in patients with schizophrenia [21].

Sixteen states legalized posttraumatic stress disorder (PTSD) as an implication and primary reason for marijuana usage; therefore, marijuana is requested from more than one-third of PTSD patients [23–26]. A synthetic form of cannabis was explored and showed significant clinical improvement in nightmares and subsidence of PTSD symptoms in patients who were non-responsive to available traditional therapies. It is still tough to make clinical recommendations for the use of marijuana for PTSD, due to the contradictory data that exist and variety of included studies in systematic reviews including; experiences, case reports, and observational studies [27]. However, other observational studies showed that it is associated with increased severity of PTSD symptoms [24]. Unfortunately, it is unknown whether and to what degree these findings can be applied to general populations; thus, more research is required.

## Implications for behavioral health

There is a stigma associated with marijuana use as a treatment, and several patients waited months or years before deciding that marijuana could be a beneficial treatment. Since the last decade till now, Middle East Muslim predominant countries are amidst regional violence and myriad sectarian conflicts contributing to rising in various mental health issues especially PTSD among war veterans [28]. Primary health care workers on these regions render treatments recommending conventional therapies to support affected populations who are experiencing traumatic events consequently leading to increased risk of developing PTSD. Marijuana can be a potential intervention to recommend on this population because cross-cultural usage of western medicine is largely different from this regional medical practice. On such background of therapeutic benefit, through treating this regional mental health burden could be lessened if researchers take initiatives to do further research work to generate concrete evidence. Although the benefits and harms of medical marijuana are still being evaluated, attitudes of physicians and other health professionals play a big role in communication with patients. Therefore, besides conducting further research in the effect of marijuana on patients’ health, more attention should also be paid to educating healthcare providers on medical marijuana treatment and implementation of an awareness program to clinicians and mass population in this affected community involving corresponding legal and religious framework could pose a favorable environment on society that may help researchers to explore influence of marijuana from mental health perspective. In addition, open discussion on the matter is important to inform the community about the impacts as well as resolving any stigmas surrounding medical marijuana.

Literature supports the proposition that marijuana and cannabinoids have substantial adverse effects on the human body’s resistance to various infections through altering immune cell function and cytokines production to invading micro-organisms [29]. Moreover, little is known about the long-term effects of marijuana on diabetes, hyperlipidemia, acute myocardial infarction, stroke, and cardiovascular mortality [30]. More confirmed prospective studies with big sample sizes are required to discover the safe zone of doses and prescription schedule time of chronic marijuana usage on CVS. Many studies proposed metabolic benefits from marijuana usage; however, mostly they were cross-sectional designs and not confirmed with prospective studies. Marijuana consumption has been known to increase appetite and food consumption [31]. Despite increased caloric intake, 15-year longitudinal data showed the extensive use of marijuana was not associated with an increase in BMI, lipid, or glucose values [7]. Increase caloric intake is likely to be mediated through the cannabinoid receptor type 1 [32]. Rimonabant, a selective blocker of the cannabinoid receptor type 1, has been used for the treatment of abdominal obesity [33]. Nevertheless, among blacks, current marijuana use has been found to be associated with significantly lower waist circumference, compared to former or never users [34]. The use also showed a tendency towards lower metabolic parameters, including total cholesterol, triglyceride, high-density lipoprotein cholesterol, and body mass index [34]. In addition, brain glucose metabolism in chronic marijuana users was studied [35]. While marijuana abusers showed decreased cerebellar metabolism at baseline compared to non-users, THC consumption increased brain glucose metabolism in the orbitofrontal cortex, prefrontal cortex, and basal ganglia in chronic users [35]. Nevertheless, in healthy individuals, chronic marijuana smoking was associated with visceral adiposity and adipose tissue insulin resistance but not with hepatic steatosis, insulin insensitivity, impaired pancreatic β-cell function, or glucose intolerance [36].

Therefore, an objective and evidence-based report of the short- and long-term health effects of marijuana use (both harmful and beneficial effects), which fully investigates the impacts of regulatory barriers to cannabis research and that proposes strategies for supporting the development of the resources and infrastructure necessary to conduct a comprehensive cannabis research agenda, is warranted.

## Abbreviations

AMI: Acute myocardial infarction
CVS: Cardiovascular system
PTSD: Posttraumatic stress disorder
